# Physiologic blood flow is turbulent

**DOI:** 10.1038/s41598-020-72309-8

**Published:** 2020-09-23

**Authors:** Khalid M. Saqr, Simon Tupin, Sherif Rashad, Toshiki Endo, Kuniyasu Niizuma, Teiji Tominaga, Makoto Ohta

**Affiliations:** 1 Biomedical Flow Dynamics Laboratory (Ohta-Lab), Institute of Fluid Science, Tohoku University, Sendai, Miyagi 980-8577 Japan; 2 Department of Neurosurgical Engineering and Translational Neuroscience, Tohoku University Graduate School of Medicine, Sendai, Miyagi 980-8574 Japan; 3 Department of Neurosurgery, Tohoku University Graduate School of Medicine, Sendai, Miyagi 980-8574 Japan; 4 Department of Neurosurgical Engineering and Translational Neuroscience, Graduate School of Biomedical Engineering, Tohoku University, Sendai, Miyagi 980-8574 Japan

**Keywords:** Circulation, Fluid dynamics

## Abstract

Contemporary paradigm of peripheral and intracranial vascular hemodynamics considers physiologic blood flow to be laminar. Transition to turbulence is considered as a driving factor for numerous diseases such as atherosclerosis, stenosis and aneurysm. Recently, turbulent flow patterns were detected in intracranial aneurysm at Reynolds number below 400 both in vitro and in silico. Blood flow is multiharmonic with considerable frequency spectra and its transition to turbulence cannot be characterized by the current transition theory of monoharmonic pulsatile flow. Thus, we decided to explore the origins of such long-standing assumption of physiologic blood flow laminarity. Here, we hypothesize that the inherited dynamics of blood flow in main arteries dictate the existence of turbulence in physiologic conditions. To illustrate our hypothesis, we have used methods and tools from chaos theory, hydrodynamic stability theory and fluid dynamics to explore the existence of turbulence in physiologic blood flow. Our investigation shows that blood flow, both as described by the Navier–Stokes equation and in vivo, exhibits three major characteristics of turbulence. Womersley’s exact solution of the Navier–Stokes equation has been used with the flow waveforms from HaeMod database, to offer reproducible evidence for our findings, as well as evidence from Doppler ultrasound measurements from healthy volunteers who are some of the authors. We evidently show that physiologic blood flow is: (1) sensitive to initial conditions, (2) in global hydrodynamic instability and (3) undergoes kinetic energy cascade of non-Kolmogorov type. We propose a novel modification of the theory of vascular hemodynamics that calls for rethinking the hemodynamic–biologic links that govern physiologic and pathologic processes.

## Introduction

The pioneering work of Womersley^1^ and his coworkers^2,3^ essentially laid the foundation of modern hemodynamics research. By developing a time-dependent one-dimensional exact solution of the Navier–Stokes equation, Womersley showed that blood flow in main arteries can be described by a Fourier decomposition of the cardiac harmonics^3,4^. This work has been later extended to account for wall elasticity^5,6^ and non-Newtonian blood viscosity^7^. The Womersley flow model (*WFM*) became a founding principle upon which modern blood hemodynamics studies are based. Researchers have assumed, based on *WFM*, that blood flow is essentially laminar, and transition to turbulence (or presence of *disturbed* flow, which is a poorly defined hemodynamic term often used in medical research) shifts the blood hemodynamics leading to the initiation of vascular diseases such as brain aneurysms or atherosclerosis^8,9^. Such disease association is attributed to the mechano-sensing properties of endothelial cells that make them responsive to various flow properties^10^. Therefore, the accurate identification of blood flow regimes is an essential step in characterizing the hemodynamic patterns that govern endothelial cells mechanobiology^9^. We have previously shown how the inaccurate assumptions of blood viscosity and the misinterpretation of wall shear stress (WSS) ^9,11^ have impacted intracranial aneurysm research, leading to inconsistently varying and contradicting results^9^. Moreover, we have recently shown that turbulence exists in pulsatile multiharmonic flow of mean Reynolds number $R{e}_{m}\sim 300$ in idealized model of intracranial aneurysm flow using particle imaging velocimetry (PIV)^12^. Similar transitional and turbulent regimes were detected in intracranial aneurysm by other research groups in vitro^13^ and in silico^14–18^. In a seminal article, Jain et al^18^ showed that at peak systolic conditions, intracranial aneurysm exhibit random velocity fluctuations and kinetic energy cascade at $Re<400$ in the parent artery. Subsequently, Jain et al^17^ argued that such complex flow patterns may alter our understanding of aneurysm growth and rupture. Turbulence casts complexity not only on the hemodynamics of intracranial aneurysm, but also on such of carotid occlusive disease^19,20^. It is established that *disturbed* flow is associated with carotid stenosis^21^ and atherosclerosis^22^. While the exact nature, characteristics and regime of such *disturbed* flow is ambiguous in literature^8,23^, numerous studies investigated its parametric relationship with the diseases progression. Kefayati et al^24^ showed that turbulence intensity varies considerably with the variation of carotid stenosis severity in vitro using particle image velocimetry. Grinberg et al.^25^ found mixed states of laminar and turbulent flows downstream the stenosis using high-resolution CFD model. Vergara et al.^26^ used Large Eddy Simulation to study transition to turbulence in abdominal aortic aneurysm. By analyzing the standard deviation of *Q-*criterion in the flow domain, they identified the transitional mechanisms that occurs within the aneurysm flow. To that end, it can be argued that the contemporary theory of non-laminar flow regimes (i.e. transitional/turbulent/disturbed) are considered to be associated with pathologic conditions while physiologic condition is believed to coincide with laminar and stable flow regime. The purpose of this article is to introduce an alternate paradigm where blood flow is seen as turbulent in both physiologic and pathologic states. However, the notion and interpretation of *turbulent flow* in this alternate paradigm are different from such of *fully developed turbulent flow* commonly perceived in literature through Reynolds criteria and Kolmogorov–Obukhov statistical theory of isotropic homogenous turbulence. This is thoroughly discussed later in this article.

**Figure 1:**
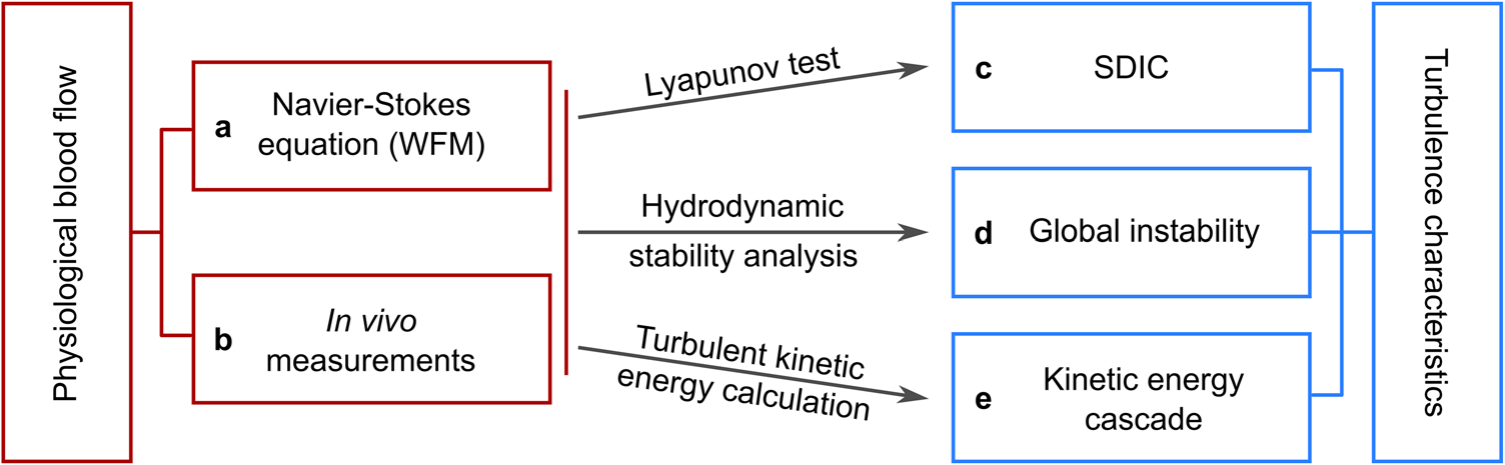
Schematic illustration of the methodology and approach used in the present work. Space–time velocity fields of physiologic blood flow were obtained from (**a**) exact solution of the Navier–Stokes equation as described by Womersley flow model with physiologically realistic boundary conditions from HaeMod database and (**b**) in vivo Doppler ultrasound measurements of healthy volunteers. Physiologic blood flow was tested for three characteristics of turbulence that are (**c**) sensitive dependence on initial conditions, (**d**) global instability and (**e**) turbulent kinetic energy cascade. Three methods were used to test such characteristics namely Lyapunov exponents test, hydrodynamic stability analysis and calculation of kinetic energy cascade in space and frequency domains.

The methodology and approach used in the present work are schematically illustrated in Fig. 1. Blood flow is represented using two sets of data: the first set is obtained from the exact solution of Navier–Stokes equation as presented in the WFM. The second set is obtained from Doppler Ultrasound (DU) measurements of the carotid artery of healthy volunteers (two of the authors). The exact solution of WFM was established using boundary conditions from the Hae-Mod open access database (http://haemod.uk/) to offer reproducible evidence of our work. The difference between the two datasets is obvious; WFM describes ideal flow while DU measurements describe real physiologic flow. By ideal we mean the flow described by WFM is one-dimensional, Newtonian, single phase and is bounded by rigid circular wall. Real physiologic flow is three-dimensional, non-Newtonian, multi-phase, and is bounded by elastic semi-circular wall. The purpose of the present work is to demonstrate that the existence of turbulence in physiologic blood flow is inherited from its simplest mathematical formulation. To that end, the purpose of using DU measurements is to show that the existence of turbulence, as shown by the exact solution, can also be demonstrated in vivo. First, Lyapunov exponents were calculated for both datasets using the open-source code provided by Wolf et al^27^. Then, criteria for hydrodynamic stability of multiharmonic pulsatile flow were derived from the Reynolds-Orr equation and used to examine the global instability of blood flow, as it is represented by both datasets. Finally, the kinetic energy cascade from both datasets was analyzed in space and frequency domains.

## Results

Here, we present the comparative evidence on the inherent turbulence in the carotid artery and we argue that similar behavior should be observed in other major arteries of the human body. Results from the exact solution of three other arteries (aortic root, thoracic aorta and iliac) are also presented. We hypothesize that the intrinsic multiharmonic waveforms of blood flow have properties that drive blood flow towards chaotic turbulent state. As much as it is important to characterize the differences between vessels in such regard, the authors believe that it would be cumbersome to conduct such characterization in this article. We invite the community to reproduce the following results using other sets of data for different arteries to further investigate inherent blood flow turbulence.

### Vascular blood flow is chaotic

**Figure 2:**
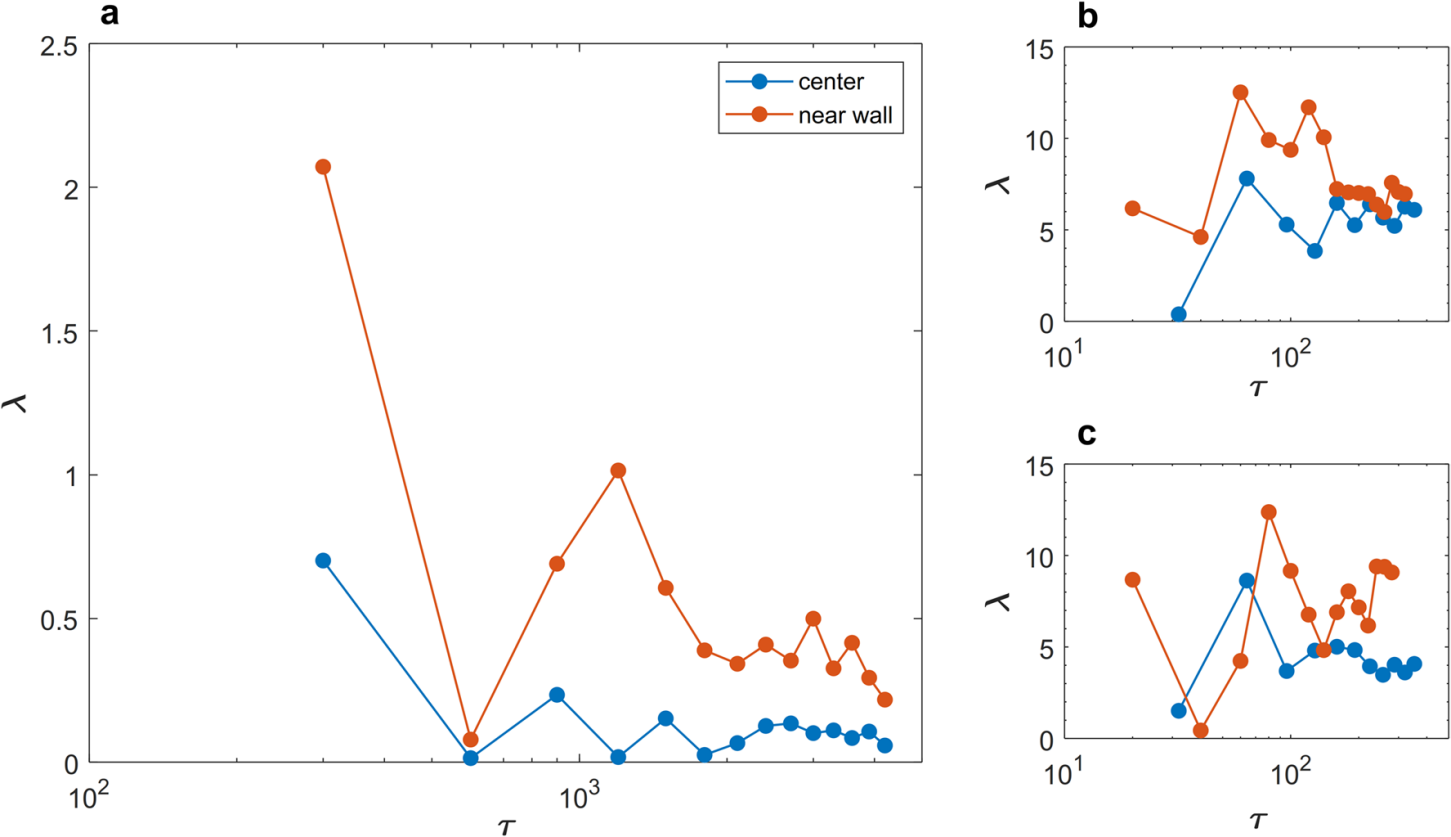
Positive Lyapunov exponents indicate SIDC in both exact solution of the Womersley equation and DU in-vivo measurements. (**a**) The orbital (τ) traces of Lyapunov exponent (λ) from exact solution at the center and 1 μm away from the wall of the Carotid artery. (**b**,**c**) Lyapunov exponents in the center and 1/3 D away from the wall of the carotid artery, respectively, in two healthy volunteers.

First, we borrowed tools from the chaos theory to test for the SDIC^27^, and to demonstrate that blood flow, expressed by both exact solution and in vivo measurements, is not periodic. We have obtained Lyapunov exponents, as a test of SDIC associated with turbulence^28^, from the time series of blood velocity obtained from the Womersley equation and from DU measurements. Figure 2a shows the trace of Lyapunov exponents in orbital space for the carotid artery. Similar Lyapunov exponents obtained from in vivo DU measurements are shown in Fig. 2b,c for two healthy volunteers (two of the authors). Positive Lyapunov exponents, indicating SDIC, has been previously reported in few studies investigating pulsatile flow in an artificial heart^29^. However, this is the first evidence on the existence of SDIC in Womersley equation. A more elaborative discussion of relevant researches on SDIC of blood flow is presented in the discussion section.

### Vascular blood flow is globally unstable

**Figure 3:**
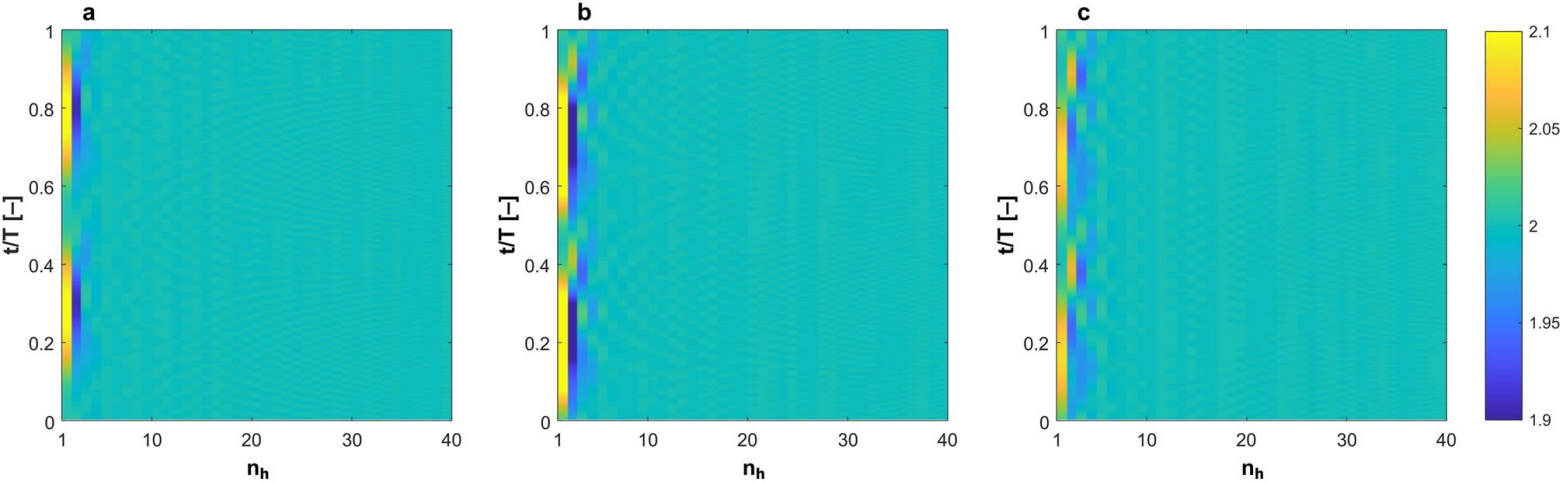
Global hydrodynamic instability condition achieved in blood flow waveforms obtained from (**a**) exact solution of Womersley equation and (**b**,**c**) in-vivo DU measurements. Harmonic number represents the number of harmonics in one cardiac cycle in both datasets and the colourmap represents the values of $\underset{t\to \infty }{\mathrm{lim}}\frac{{E}_{V}(n)}{{E}_{V}(n-1)}\ne 0$ corresponding to 40 harmonics which constitute the pulsatile waveform.

**Figure 4:**
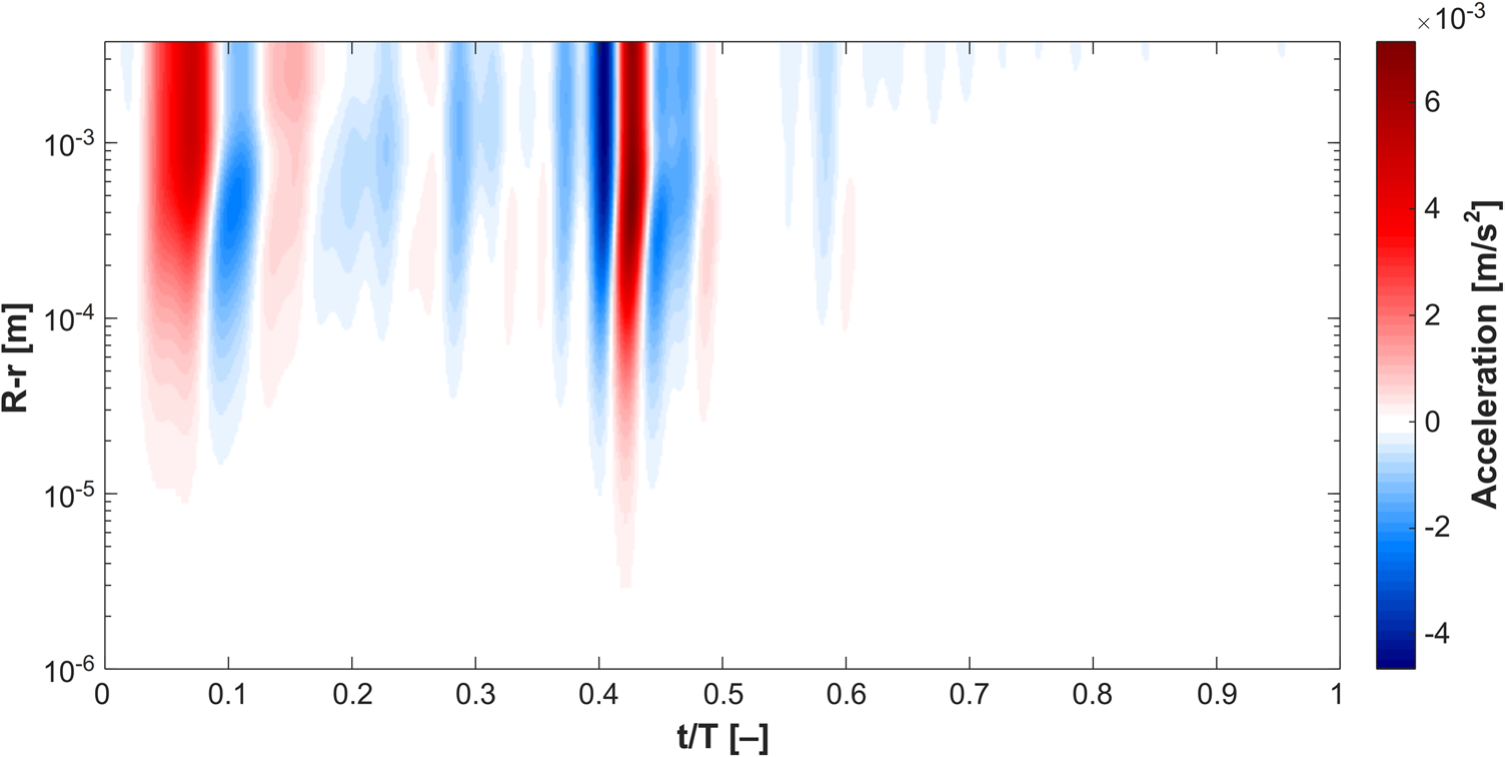
Acceleration field obtained from the Womersley exact solution. The field is dominated by quasi-steady flow $\frac{\partial v}{\partial t}\approx 0$ while peaks of minimal and maximal acceleration reciprocate at distance corresponding to $50 \mathrm{\mu m}$ from the wall. Acceleration alters the flow energy significantly driving it towards global instability.

The theory of hydrodynamic stability has been developed to evaluate the stability of steady flow under finite space–time perturbation^30,31^. Some works have evaluated the stability of monoharmonic (i.e. sinusoidal) flow^32–36^. However, there are no universal criteria that can generally describe the stability of multiharmonic pulsatile flow. We have extended the available criteria to evaluate Womersley flow. In Womersley flow, the velocity field is given as:

1 $$u\left({x}_{i},t\right)={u}_{o}\left({x}_{i}\right)+\sum_{i=1}^{n}{a}_{i}\mathrm{sin}\left(i\omega t\right)+{b}_{i}\mathrm{cos}\left(i\omega t\right)$$

where $n$ is the total number of harmonics in a specific waveform. Here, we propose that a given Womersley flow is globally unstable if $\underset{t\to \infty }{\mathrm{lim}}\frac{{E}_{V}(n)}{{E}_{V}(n-1)}\ne 0$. Figure 3 shows colourmap of such condition as obtained both from exact solution and in vivo DU measurements of the carotid artery. It is clear that the condition is achieved in both datasets confirming that blood flow is globally unstable. In Fig. 4, the acceleration field obtained from the exact solution is depicted. Maximum acceleration and minimum deceleration are proximal in time domain, while the remaining time domain of the acceleration field is dominated by quasi-steady flow. This supports the assumption made to evaluate the global instability, bearing in mind that ${\int }_{0}^{\infty }{E}_{V}dV=\frac{1}{2}\stackrel{-}{{\tilde{v}}^{2}{({x}_{i}, t)}}$ is primarily affected by convective acceleration $\overrightarrow{v}\bullet \nabla \overrightarrow{v}$, where $\tilde{v}$ is the instantaneous velocity field.

### Physiologic vascular blood flow exhibits kinetic energy cascade

**Figure 5:**
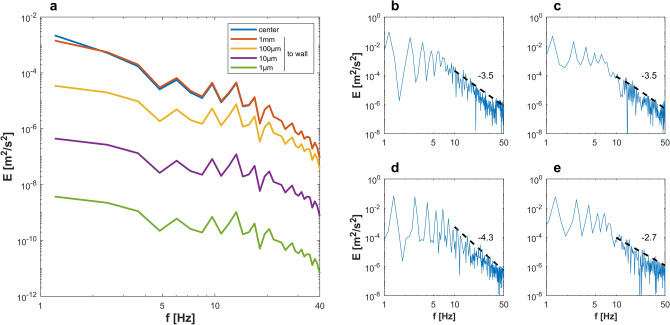
Kinetic energy cascade detects non-Kolmogorov kinetic energy transfer at frequencies higher than dominant frequency. In (**a**), the kinetic energy cascade obtained from the exact solution of Womersley flow of carotid artery are plotted at varying locations from the wall. Similar cascades are displayed for the carotid blood flow of two healthy volunteers at the artery center (**b**,**d**) and at $\frac{1}{3}D$ away from the wall (**c**,**e**). The scaling of energy cascades is found to be (**b**,**c**) $-\frac{7}{2}$ (**d**) $-\frac{43}{10}$ (**e**) $-\frac{27}{10}$.

One of the most profound properties of turbulence is the cascade of kinetic energy. Kinetic energy is transferred from larger to smaller vortex structures and ends up irreversibly dissipating to the surrounding media in the form of heat. Figure 5 shows kinetic energy cascade in carotid blood flow as depicted from exact solution and in vivo DU measurements. To the best of the authors’ knowledge, the depicted slopes of such cascade do not match any of the Kolmogorov scales reported in literature. Turbulence, in such case, is of non-Kolmogorov regime. Such uncharacteristic turbulence regime has been observed in atmospheric turbulence^37,38^, however, it is the first time to be reported in this physiologic setting. Until the present moment, the main mechanism linking hemodynamics to biological and pathological processes in arteries is the wall shear stress^8^. The existence of kinetic energy cascade in blood flow should develop this mechanism to include other mechanical and kinetic factors that could contribute to mechanosensory and mechanotransduction of endothelial cells.

## Discussion

In fluid mechanics, laminar flow is a special case in which the nonlinearity of Navier–Stokes equation is far less than such of the general case of turbulent flow. It is difficult to trace the origins of blood flow laminarity assumption in the early works of Womersley et al. However, it is generally believed that since the *WFM* describes quasi-periodic flow in cylindrical conduits (i.e. vessels) with Reynolds number below 1,000, it would not fit into the Kolmogorov–Obukhov statistical theory of turbulence. The Reynolds criteria was used to classify vascular blood flow, although its essential assumptions (i.e. steadiness, Newtonian viscosity and uniform geometry) do not apply on blood flow. Hence, the classification of Womersley flow was established to be laminar flow in normal human arteries^39,40^. In the following decades, all the vascular diseases models were established based on that classification. The deviation from the so called laminar flow conditions is believed to be linked to atherogenesis and aneurysm formation through various mechanisms associated with endothelial cells inflammatory reactions and dysfunction^8,41–44^. This reasoning has dominated vascular biology research, and most of the body of literature regarding endothelial cells response to hemodynamics for the past four decades^8,45^.

The notion of turbulence in relevant literature often refers to Reynold’s *turbulent flow criteria*^46,47^ that describes *fully developed turbulent flow*. The associated interpretation of *turbulence* is, therefore, limited to isotropic homogenous turbulence. Kolmogorov^48,49^ and Obukhov^50,51^ proposed the statistical theory of isotropic homogenous turbulence based on fully developed turbulent flow^52^. Such theory is derived based on key assumptions. Taylor’s frozen turbulence hypothesis^53^ is one example of these assumptions. It relates spatial statistics to temporal statistics and holds only if $\tilde{u}/U\ll 1$. In our work, such ratio has larger ranges, as shown in Supplementary Figure S1. Therefore, the notion of *turbulence* in our work indicates turbulent flow that does not fall within the Kolmogorov–Obukhov theory. Subsequently, we have borrowed the expression *non-Kolmogorov turbulence*^54,55^ from atmospheric turbulence literature to describe our observations. Despite the differences between the former and the latter, both phenomena cannot be described by Kolmogorov–Obukhov theory.

**Figure 6:**
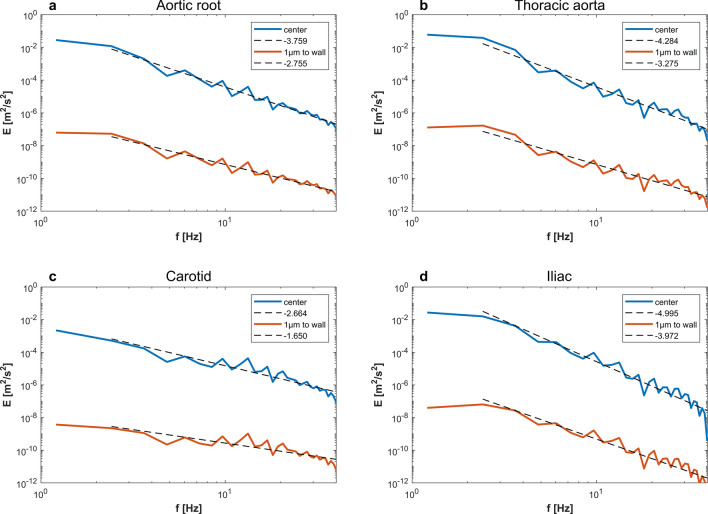
Kinetic energy cascade calculated from the Womersley exact solution. Solid lines represent the energy cascade at the center (blue) and $1\mathrm{ \mu m}$ from the wall (red) and dashed lines represent the log fitting to show the slope of the cascade, based on the boundary conditions representing (**a**) aortic root [approx. slope:$-\frac{15}{4},-\frac{8}{3}$] (**b**) thoracic aorta [approx. slope:$-\frac{43}{10},-\frac{10}{3}$] (**c**) carotid artery [approx. slope:$-\frac{13}{5},-\frac{5}{3}$] and (**d**) iliac artery [approx. slope:$-5,-4$].

Turbulent flow has many characteristics that keeps it as one of the standing mysteries in classical physics^56^. Some of these characteristics are chaos, instability, and kinetic energy cascade^57^. Turbulent flow is sensitive to initial conditions, which makes it subjected to the properties of chaos theory^58^. A turbulent field is instable where any finite perturbation in space and time propagates. The vorticity field is always non-zero in turbulent flows, and the kinetic energy transfers from large to small vortices and vice versa^59^. The cascade of kinetic energy is a characteristic feature of turbulent flows and it contributes to the very definition of turbulence^60,61^. Steady, periodic and quasi-periodic laminar flows tend to conserve their kinetic energy due to the absence of the vortex formation and breakup phenomena. The classical Kolmogorov–Obukhov theory of turbulence describes isotropic homogenous turbulence in which energy cascade is subjected to scaling laws that define the rate of transfer of kinetic energy^62^. Figure 6 shows the kinetic energy cascade, in frequency domain, for four arteries at two different radial locations based on the WFM exact solution. It is clear from Fig. 6 that the slope of kinetic energy cascade is affected by spatial location (i.e. radial distance from the wall). This is further explained in Supplementary Figure S2, where the radial profiles of kinetic energy cascade slope are plotted. Supplementary Figure S2 shows that the slopes vary with radius, in the four arteries, with values close to Kolmogorov scaling laws $(\frac{p}{3})$ near the artery wall and non-Kolmogorov values in the proximity of the arteries’ centerline. It is clear that the values of energy cascade slope are different from such commonly observed in the classical theory of turbulence^63,64^. It is also important to mention that non-Kolmogorov turbulence was detected in carotid artery stenosis by Lancellotti et al. using large eddy simulation^65^ and Mancini et al. using direct numerical simulation (DNS) and LES^66^. Here we show that it exists in physiological conditions as described by the governing equation and in vivo measurements.

Chaotic behavior, as observed in SDIC of blood flow, has been a focal interest of some studies along the past three decades. Yip et al^67^ found positive Lyapunov exponents in the renal artery of spontaneously hypertensive rats (SHR). Their results indicated the presence of a low-dimension strange attractor. While studying artificial heart systems, Yambe et al.^68^ discovered that blood flow waveforms have positive Lyapunov exponents and investigated the phase portraits of the dynamical system composed by the flow waveform. They demonstrated the existence of lower dimensional chaotic dynamics in blood flow. The works of Yin et al. and Yambe et al. used multiharmonic flow functions of the same form used in the present work (Eq. 1), however, their estimation of Lyapunov exponent was space-independent. When polynomial functions are used to describe blood flow waveforms, such as in the works of Bračič and Stefanovska^69,70^, Lyapunov exponents come in pairs showing the periodic nature of these functions. Also, the level of details of the harmonic function is crucial to describe the actual physics of the flow. Here, he have used 40 harmonics to compose the solution of Eq. (1). We argue here that positive Lyapunov exponents, as an indicator of SDIC, are intrinsic properties of the Womersley equation given that sufficient number of harmonics is used to describe blood flow accurately. This is inductively shown by the in vivo data. Visee et al.^71^ used consecutive transcranial Doppler (TCD) to investigate the existence of chaos in patients with occlusive cerebrovascular disease. They had evidently shown that blood flow in healthy conditions exhibited positive Lyapunov exponents, while in impaired intracranial circulation it was found to be of periodic nature with near-zero Lyapunov exponents. These findings have been also supported by the works of Ozturk^72^ and Ozturk and Arslan^73^ where TCD signals showed positive Lyapunov exponents in the intracranial circulation. In our analysis of the Womersley equation and in vivo DU measurements, we performed Lyapunov exponent analysis, which essentially informs us whether the data or equation are inherently chaotic or not. Womersley equation had a positive Lyapunov exponent, meaning that it has properties of chaos under multiharmonic pulsatile flow that is the blood flow. This, by definition, negates the assumption that the *WFM* describes a pulsatile laminar blood flow regime. Thus, we argue that a transition to turbulence per se is not a cause for disease initiation, rather a change in turbulence characteristics due to complex geometry or harmonics would be the culprit. This of course adds a layer of complexity to vascular hemodynamics and biology.

**Figure 7:**
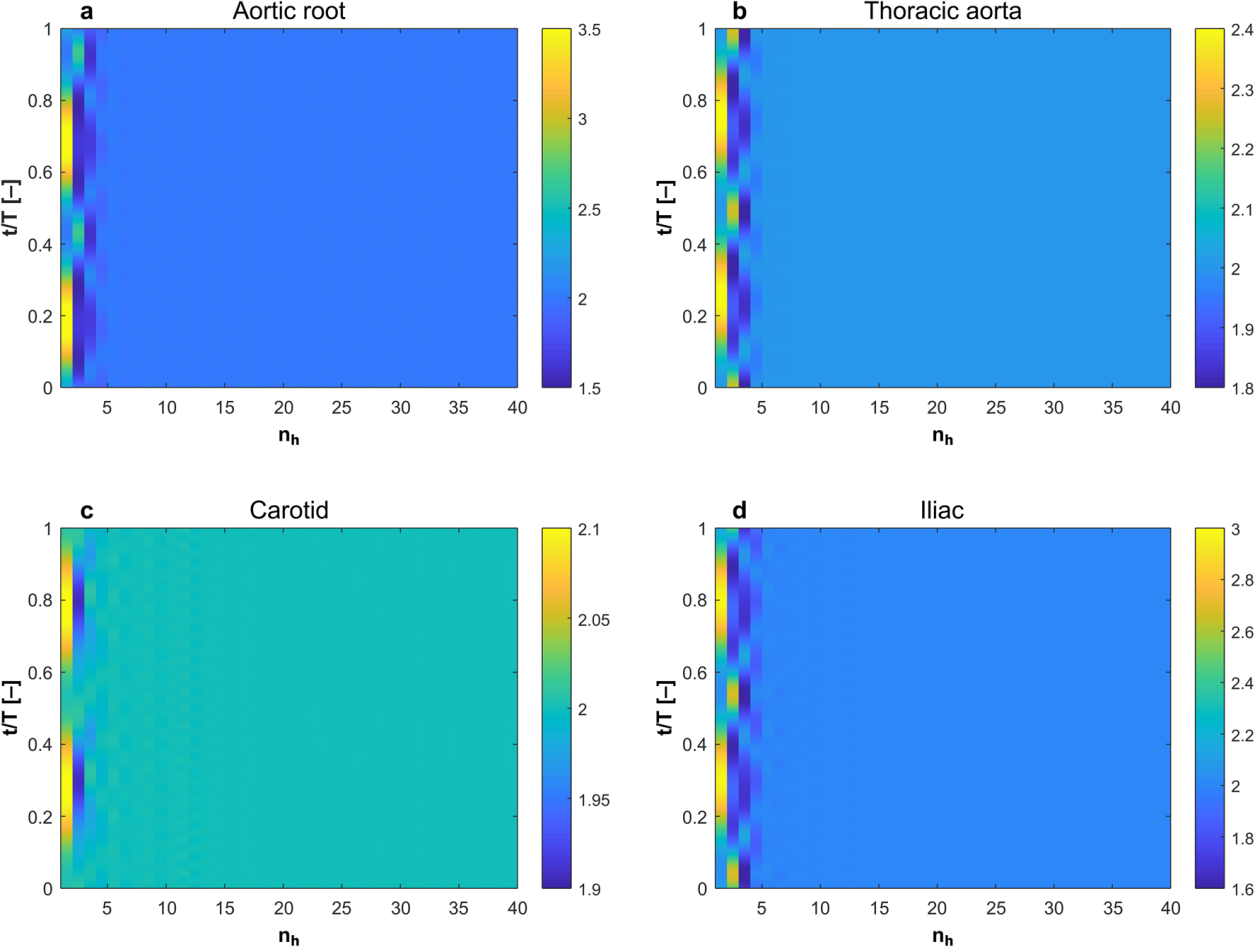
Colourmaps of $\lim\nolimits_{n \to \infty }{\mathrm{lim}}\frac{{E}_{V}(n)}{{E}_{V}(n-1)}\ne 0$ in from the exact solution of Womersley flow equation in (**a**) aortic root (**b**) thoracic aorta (**c**) carotid artery and (**d**) iliac artery.

In fluid mechanics, it is generally believed that hydrodynamic instability is always associated with turbulence^74^. However, most of the work done to understand the correlation between the two phenomena in pipe flow was mostly limited to steady flows subjected to finite perturbations in space and time^75^. The stability of pulsatile flows have been studied in some works^76–79^, however, there is no consensual methodology among fluid dynamicists for investigating their stability. Therefore, to study the global instability of blood flow both from Womersley equation and in vivo measurements, we had to adopt the main principles of the hydrodynamic stability theory and develop intuitive representation of its criteria for global instability. The concept underlying this approach is fairly simple. Serrin^80^ argued that for a viscous flow to be considered stable *“the energy of any disturbance tends to zero as* $t$*increases”*. Hence, we assumed that each harmonic component of the flow waveform $(n)$ could be viewed as a space–time perturbation to its predecessor in in frequency domain $(n-1)$, as shown in Fig. 3. In Fig. 7, the stability condition derived from this principle is plotted for four arteries based on the exact solution of Womersley equation using boundary conditions from HaeMod database. We have used 40 harmonics to represent the solution of Womersley equation. The reasoning behind this is based on a mass transfer analysis. We have analyzed the contribution of each harmonic in mass transfer by computing the mass flow rate, per unit density, $\dot{m}=A\times u({x}_{i},t)$ with a loop that changes the number of harmonics $(n)$ from 1 to 1,000. We have found that $\lim\nolimits_{n \to \infty }^{}\frac{{\dot{m}}_{n+1}-{\dot{m}}_{n}}{{\dot{m}}_{n}}=0$. Supplementary Figure S3 shows this difference for the first 40 harmonics. Based on dimensional analysis, we assume that $\lim\nolimits_{n \to 40}\left|\frac{{\dot{m}}_{t}-{\dot{m}}_{[0:n]}}{{\dot{m}}_{t}}\right|={10}^{-4}$ is an appropriate cut threshold, therefore, we have considered the first 40 waveform harmonics only.

When one thinks of laminar pulsatile flow, the only available flow field variable relevant to physiological vessel changes is wall shear stress, which is well studied and characterized especially in in vitro endothelial flow exposure experimentation^10^. On the other hand, when turbulent flow is considered, numerous variables could affect the vessel on multiple scales. For instance, kinetic energy, represented by the energy cascade, transfers from large coherent vortices to small isotropic homogenous vortices before dissipating in viscosity scales as heat. We have evaluated the length scales and energy scales of such vortices and they fall well within the limits of cellular mechano-sensing^81^. This begets an important question, how do different energy cascades, for example a direct versus an inverse cascade, impact endothelial cells? and more importantly, how can we fine tune our experimental setups to measure and reproduce these conditions? Moreover, how would these new findings impact vascular hemodynamics based therapeutic such as shear driven drug delivery, a possible drug delivery method to areas of altered shear stress^82^, or intravascular devices^83^? All of these questions, indeed, are interesting prospects of the presented results.

The main obstacle in trying to answer these questions from the available literature, is that up to this point, turbulent flow (referred to in most of the literature as disturbed flow) is considered to be pathologic as opposed to laminar flow^84,85^. Moreover, on most studies that discussed the differences between laminar and turbulent flow regimes on endothelial mechanobiology, both laminar and turbulent flows were at different WSS values^8,86,87^. Given the fact that WSS is one characteristic of flow, and the complexity of turbulent regimes, making a change of WSS impactful within various aspects of the turbulent flow that might in turn impact endothelial mechano-sensing via unidentified mechanisms, it is difficult to draw direct comparisons between the impact of laminar and turbulent flows starting from the assumption that turbulent flow itself is physiologic in healthy carotid arteries in absence of stenosis as we propose herein. To further elaborate on this notion, we previously performed a study on endothelial cells using laminar flow only at various WSS values and analyzed the microRNA expression using microarray^10^. To our surprise, we observed new unreported microRNAs to be differentially regulated between high and low WSS conditions, while previously reported microRNAs were not significant in our analysis. When we consider that most literature studying endothelial microRNA in flow responses uses turbulent flow at lower WSS compared with laminar flow at physiologic WSS^45^, the importance of performing parametric studies to characterize the differences between turbulent and laminar flow at same or similar WSS becomes obvious.

In conclusion, this work aims to propose a modification of the theory of vascular hemodynamics. That is, the blood flow is inherently turbulent and not laminar, and changes in turbulence kinetic energy or other properties of turbulence could be the driving factors behind the hemodynamics-biology links. These results and their theoretical consequences should motivate the scientific community to update their thinking regarding hemodynamic drivers of endothelial and vascular processes, given the inherent complexities and chaos associated with turbulence. Moreover, more hemodynamic research, with accurate and rigorous methodologies, should aim at further characterizing the interesting features of turbulent blood flow in physiology and pathology. To improve the accuracy of WFM, similar analysis should also be conducted with mathematical and computational models that accounts for non-Newtonian effects, platelet and blood cells dynamics and fluid–solid interaction with arterial wall.

## Materials and methods

For the sake of reproducibility, we have worked with very simple tools including a well-documented exact solution methodology of the Womersley equation, open-source physiological flow database, and open-source chaos analysis code and well-established methods of computing hydrodynamic instability and turbulent energy cascade. This section describes the details of these methods to provide the essential steps to reproduce our results. The healthy subjects’ doppler ultrasound measurements were collected from co-authors of this article who volunteered their data. No institutional approval was required as per Tohoku University’s ethical board.

### Exact solution of the Womersley equation

The Womersley equation that defines the velocity profile of any physiological flow in arteries can be written as:

2 $$\tilde{u}\left(r,t\right)=\frac{i{k}_{s}{r}^{2}}{\mu {\Omega }^{2}}\left(1-\frac{{J}_{0}\left(\zeta \right)}{{J}_{0}\left(\Lambda \right)}\right){e}^{i\omega t}$$

where ${k}_{s}{e}^{i\omega t}$ is the oscillating pressure gradient, $r$ is the artery radius, $\mu$ is the viscosity of blood, $\Omega =r\sqrt{\frac{\rho \omega }{\mu }}$ is the Womersley number, ${J}_{0}$ is the Bessel function of zero order and first kind, $\Lambda =\Omega \left(\frac{i-1}{\sqrt{2}}\right)$ is the complex frequency parameter, and $\zeta \left(r\right)=\Lambda \frac{r}{R}$ is the complex variable.

The derivation of Eq. (2) from Navier–Stokes equation can be found in the original paper by Womersley^4^ or and in more instructive details in^88^. The exact solution of Eq. (2) as initial-boundary value problem has been extensively reported in literature, however, with no evidence of checking the existence of turbulence and chaos. In this work, the initial-boundary conditions were obtained from the open-access database HaeMod (https://haemod.uk/virtual-database). This database provides blood flow waveforms for 11 major arteries. The present work establishes the investigation on the carotid artery (using the database as well as via doppler U/S measurements from volunteers) and provides the exact solution results for three more arteries in supplementary materials. The waveforms were created using physiologically realistic model based on human data. HaeMod database has been extensively validated in literature and confirmed to represent physiologically relevant and valuable data. However, Fourier decomposition revealed that the waveforms are limited to 40 harmonics. No institutional approval was required for the DU measurements as per Tohoku University and Kohnan hospital (the location of the DU measurements) ethics guidelines.

### Lyapunov exponent calculations

For any time series dataset, the rate of separation of infinitesimally close orbital trajectories are characterized by Lyapunov exponent where the initial separation is $\delta {X}_{o}$ and the divergence rate is $\left|\delta X(t)\right|\approx {e}^{\lambda t}\left|\delta {X}_{o}\right|$ where $t$ is time and $\lambda$ is the Lyapunov exponent. We have used the open-source code provided by Wolf et al.^89^ and described in their 1985 famous paper^27^ to build the phase-space and orbits of the time series datasets obtained from the exact solution of the Womersley equation and DU measurements.

### Evaluation of global hydrodynamic stability in pulsatile flow

The onset of transition in physiological flow requires hydrodynamic instability to commence. Such instability is caused by a sustained disturbance in the flow field, where its energy is expressed as:

3 $${\int }_{0}^{\infty }{E}_{V}dV=\frac{1}{2}\stackrel{-}{{\tilde{v}}^{2}{({x}_{i}, t)}}$$

where $\tilde{v}\left({x}_{i},t\right)\notin u\left({x}_{i},t\right)={u}_{o}({x}_{i})+\sum_{i=1}^{\mathrm{n}}{a}_{i}\mathrm{cos}(i\omega t)+{b}_{i}\mathrm{sin}(i\omega t)$ where $u({x}_{i},t)$ is the blood velocity waveform with ${a}_{i}$ and ${b}_{i}$ as the Fourier coefficients of the Womersley waveform, and ${u}_{o}({x}_{i})$ is the steady component of the flow which is characterized by Hagen–Poiseuille parabolic velocity profile. The sustenance of a disturbance must be global (i.e. in space and time) for transition to take place.

In order for the flow to become globally instable, the time rate of change of ${E}_{V}$ must be positive, hence the disturbance can possibly prevail over the viscous resistance of the fluid. The Reynolds–Orr equation^90^ for disturbance energy reads:

4 $$\frac{d{E}_{V}}{dt}=-\underset{V}{\overset{}{\int }}u\cdot \left({S}_{ij}u\right)dV- \frac{2}{Re}\underset{V}{\overset{}{\int }}{s}_{ij}:{s}_{ij}dV$$

where *t* is time, $S_{ij}$ and $s_{ij}$ are the strain rates of basic and altered (i.e. disturbed) flows, respectively, $Re$ is the Reynolds number and $V$ is the wall-bounded flow volume. The first term on the right-hand side (RHS) of (3) describes the exchange of energy with the basic flow, while the second term describes the energy dissipation due to viscosity. If the former is higher than the latter (i.e. the disturbance energy does not decay with time), the flow will be globally instable. Thus, the criteria for global stability can be written in terms of critical Reynolds number as:

5 $$\frac{1}{{Re}_{cr,m}}=\underset{v\left(x,t\right)=0}{\mathrm{max}}\frac{-{\int }_{V}u\bullet \left({S}_{ij}u\right)dV}{2{\int }_{V}{s}_{ij}:{s}_{ij}dV} when\frac{d{E}_{V}}{dt}=0$$

In Eq. (5), the critical Reynolds number ${Re}_{cr,m}$ defines the global and monotonic stability criterion for viscous fluid flow subjected to a disturbance of the velocity $u(x,t)$. Hence, if $Re>{Re}_{cr,m}$ the flow becomes monotonically instable, in other words the disturbance will not decay for $t \to \infty$ and transition will take place. Hence, asymptotic instability occurs when $\lim\nolimits_{n \to \infty }\frac{{E}_{V}(t)}{{E}_{V}(0)}\ne 0$. In pulsatile multiharmonic flow, such condition takes the form $\lim\nolimits_{n \to \infty }\frac{{E}_{V}(n)}{{E}_{V}(n-1)}\ne 0$ where $n$ is the index of harmonic in the Womersley velocity waveform described in Eq. (3). This condition has been evaluated in space and frequency domain using Eq. (3) and the trapezoidal method for numerical differentiation. Following Thomas^91,92^ and Hopf^93^, Serrin^80^ proved that in order to have monotonic instability in any viscous flow, the disturbance energy must satisfy the following condition:

6 $${E}_{V}>{E}_{VO} {e}^{\frac{t}{v}\left({U}_{cr,m}^{2}-R{e}_{cr,m}\frac{{\nu }^{2}}{{d}^{2}}\right)}$$

where ${E}_{VO}$ is the initial disturbance kinetic energy, ${U}_{mc}$ is the velocity of the basic flow during a one cycle of the pulsatile flow. Serrin’s second theorem proved that ${Re}_{cr,m}=32.6$ for viscous incompressible wall bounded flows. Reynolds number in main arteries is larger than the latter value. Hence, blood flow, by definition, is monotonically instable.

### Calculating energy cascade in frequency domain

**Figure 8:**
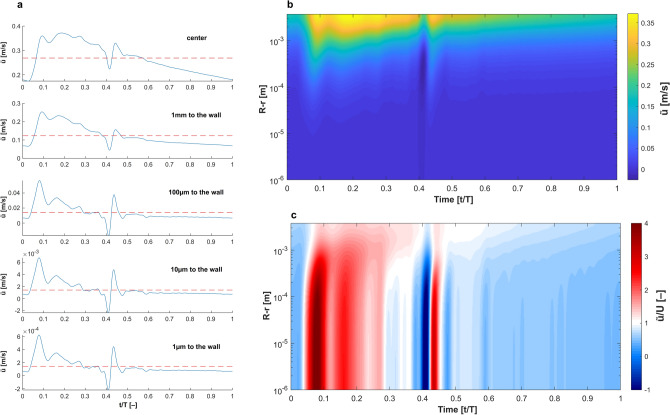
The advection of oscillating harmonics in carotid waveform cannot be correlated with the mean flow velocity as long as $\frac{\tilde{u}}{U}>1$, hence, it is not possible to obtain the energy field in wavenumber domain. (**a**) The carotid waveform at varying distance from the wall is plotted against dimensionless time and the red dashed line depicts the mean flow velocity. The flow near the wall is more complex than such away from the wall. (**b**) Colourmap of the velocity field of one cycle plotted against distance from the wall. (**c**) Colourmap of $\frac{\tilde{u}}{U}$ plotted against distance from the wall.

The local kinetic energy in frequency domain was calculated as^94^: ${E}_{i}\left(f\right)=\frac{{\left|\mathcal{F}\left\{{\tilde{u}}_{i}\left(t\right)\right\}\right|}^{2}}{L\bullet f}$ where $f$ is the frequency, $\mathcal{F}$ is the fast Fourier transform and L the length of ${\tilde{u}}_{i}$ matrix. It was not possible to use Taylor’s hypothesis to calculate the kinetic energy in wavenumber domain since $\frac{\tilde{u}}{U}>1$. Figure 8 shows velocity patterns in radial and time domains of the carotid artery to show that the conditions for using Taylor’s hypothesis do not apply.

## Supplementary information

Supplementary information.

## Data Availability

Raw data required to reproduce the carotid artery analysis based on WFM has been deposited at Harvard Dataverse and is available for public access under CC license at: https://doi.org/10.7910/DVN/OM1T42

## References

[1] Womersley JR (1954). Flow in the larger arteries and its relation to the oscillating pressure. J. Physiol..

[2] Hale JF, McDonald DA, Taylor MG, Womersley JR (1955). The counter chronometer method for recording pulse-wave velocity. J. Physiol..

[3] Hale JF, McDonald DA, Womersley JR (1955). Velocity profiles of oscillating arterial flow, with some calculations of viscous drag and the Reynolds number. J. Physiol..

[4] Womersley JR (1955). Method for the calculation of velocity, rate of flow and viscous drag in arteries when the pressure gradient is known. J. Physiol..

[5] Womersley JR (1955). Mathematical theory of oscillating flow in an elastic tube. J. Physiol..

[6] Womersley JR (1957). Oscillatory flow in arteries: The constrained elastic tube as a model of arterial flow and pulse transmission. Phys. Med. Biol..

[7] Womersley JR (1958). Oscillatory flow in arteries. III: Flow and pulse-velocity formulae for a liquid whose viscosity varies with frequency. Phys. Med. Biol..

[8] Chiu JJ, Chien S (2011). Effects of disturbed flow on vascular endothelium: Pathophysiological basis and clinical perspectives. Physiol. Rev..

[9] Saqr KM (2019). What does computational fluid dynamics tell us about intracranial aneurysms? A meta-analysis and critical review. J. Cereb. Blood Flow Metab..

[10] Rashad S (2020). Epigenetic response of endothelial cells to different wall shear stress magnitudes: A report of new mechano-miRNAs. J. Cell. Physiol..

[11] Saqr KM (2019). Wall shear stress in the Navier–Stokes equation: A commentary. Comput. Biol. Med..

[12] Tupin, S. *et al.* Non-Kolmogorov Turbulence and Inverse Energy Cascade in Intracranial Aneurysm: Near-Wall Scales Suggest Mechanobiological Relevance****. arXiv:2001.08234 (2020) **(arXiv preprint)**.

[13] Yagi T (2013). Experimental insights into flow impingement in cerebral aneurysm by stereoscopic particle image velocimetry: Transition from a laminar regime. J. R. Soc. Interface.

[14] Berg, P., Abdelsamie, A., Janiga, G. & Thévenin, D. in *International Symposium on Turbulence and Shear Flow Phenomena, TSFP 2013*

[15] Valen-Sendstad K, Mardal KA, Mortensen M, Reif BAP, Langtangen HP (2011). Direct numerical simulation of transitional flow in a patient-specific intracranial aneurysm. J. Biomech..

[16] Jain, K. Transition to Turbulence in Physiological Flows: Direct Numerical Simulation of Hemodynamics in Intracranial Aneurysms and Cerebrospinal Fluid Hydrodynamics in the Spinal Canal. *PhD Thesis* (universi-Universitätsverlag Siegen, 2016).

[17] Jain K, Jiang J, Strother C, Mardal KA (2016). Transitional hemodynamics in intracranial aneurysms—comparative velocity investigations with high resolution lattice Boltzmann simulations, normal resolution ANSYS simulations, and MR imaging. Med. Phys..

[18] Jain K, Roller S, Mardal KA (2016). Transitional flow in intracranial aneurysms—a space and time refinement study below the Kolmogorov scales using Lattice Boltzmann Method. Comput. Fluids.

[19] Lee SW, Antiga L, Spence JD, Steinman DA (2008). Geometry of the carotid bifurcation predicts its exposure to disturbed flow. Stroke.

[20] Blackshear WM (1979). Detection of carotid occlusive disease by ultrasonic imaging and pulsed Doppler spectrum analysis. Surgery.

[21] Toole JF, Castaldo JE (1994). Accurate measurement of carotid stenosis. J. Neuroimaging.

[22] Nam D (2009). Partial carotid ligation is a model of acutely induced disturbed flow, leading to rapid endothelial dysfunction and atherosclerosis. Am. J. Physiol-Heart C.

[23] Himburg HA, Friedman MH (2006). Correspondence of low mean shear and high harmonic content in the porcine iliac arteries. J. Biomech. Eng..

[24] Kefayati S, Holdsworth DW, Poepping TL (2014). Turbulence intensity measurements using particle image velocimetry in diseased carotid artery models: Effect of stenosis severity, plaque eccentricity, and ulceration. J. Biomech..

[25] Grinberg L, Yakhot A, Karniadakis GE (2009). Analyzing transient turbulence in a stenosed carotid artery by proper orthogonal decomposition. Ann. Biomed. Eng..

[26] Vergara C, Le Van D, Quadrio M, Formaggia L, Domanin M (2017). Large eddy simulations of blood dynamics in abdominal aortic aneurysms. Med. Eng. Phys..

[27] Wolf A, Swift JB, Swinney HL, Vastano JA (1985). Determining Lyapunov exponents from a time series. Phys. D..

[28] Faisst H, Eckhardt B (2004). Sensitive dependence on initial conditions in transition to turbulence in pipe flow. J. Fluid Mech..

[29] Yambe T (1995). Deterministic chaos in the hemodynamics of an artificial heart. ASAIO J. (Am. Soc. Artif. Internal Organs: 1992).

[30] Kerswell RR (2005). Recent progress in understanding the trasnsition to turbulence in a pipe. Nonlinearity.

[31] Darbyshire AG, Mullin T (1995). Transition to turbulence in constant-mass-flux pipe flow. J. Fluid Mech..

[32] Einav S, Sokolov M (1993). An experimental study of pulsatile pipe flow in the transition range. J. Biomech. Eng..

[33] Özdinç Çarpinlioǧlu M, Yaşar Gündoǧdu M (2001). A critical review on pulsatile pipe flow studies directing towards future research topics. Flow Meas. Instrum..

[34] Trip R, Kuik DJ, Westerweel J, Poelma C (2012). An experimental study of transitional pulsatile pipe flow. Phys. Fluids.

[35] Pier B, Schmid PJ (2017). Linear and nonlinear dynamics of pulsatile channel flow. J. Fluid Mech..

[36] Xu D, Avila M (2018). The effect of pulsation frequency on transition in pulsatile pipe flow. J. Fluid Mech..

[37] Stribling, B. E., Welsh, B. M. & Roggemann, M. C. in *Atmospheric Propagation and Remote Sensing IV.* 181–197 (International Society for Optics and Photonics).

[38] Belen'kii, M. S., Karis, S. J., Osmon, C. L., Brown, J. M. & Fugate, R. Q. in *18th Congress of the International Commission for Optics.* 50–52 (International Society for Optics and Photonics).

[39] Moore JE, Xu C, Glagov S, Zarins CK, Ku DN (1994). Fluid wall shear stress measurements in a model of the human abdominal aorta: Oscillatory behavior and relationship to atherosclerosis. Atherosclerosis.

[40] Ku DN (1997). Blood flow in arteries. Annu. Rev. Fluid Mech..

[41] Noris M (1995). Nitric oxide synthesis by cultured endothelial cells is modulated by flow conditions. Circ. Res..

[42] De Keulenaer GW (1998). Oscillatory and steady laminar shear stress differentially affect human endothelial redox state: Role of a superoxide-producing NADH oxidase. Circ. Res..

[43] Tedgui A, Mallat Z (2001). Anti-inflammatory mechanisms in the vascular wall. Circ. Res..

[44] Shyy JYJ, Chien S (2002). Role of integrins in endothelial mechanosensing of shear stress. Circ. Res..

[45] Donaldson CJ, Lao KH, Zeng L (2018). The salient role of microRNAs in atherogenesis. J. Mol. Cell. Cardiol..

[46] Jackson D, Launder B (2007). Osborne Reynolds and the publication of his papers on turbulent flow. Annu. Rev. Fluid Mech..

[47] Reynolds OXXIX (1883). An experimental investigation of the circumstances which determine whether the motion of water shall be direct or sinuous, and of the law of resistance in parallel channels. Philos. Trans. R. Soc. A..

[48] Kolmogorov AN (2006). A refinement of previous hypotheses concerning the local structure of turbulence in a viscous incompressible fluid at high Reynolds number. J. Fluid Mech..

[49] Kolmogorov AN (1991). Dissipation of energy in locally isotropic turbulence. Akad. Nauk SSSR Doklady..

[50] Oboukhov AM (1962). Some specific features of atmospheric tubulence. J. Fluid Mech..

[51] Obukhov A (1941). On the distribution of energy in the spectrum of turbulent flow. Bull. Acad. Sci. USSR Geog. Geophys..

[52] Birnir B (2013). The Kolmogorov–Obukhov statistical theory of turbulence. J. Nonlinear Sci..

[53] Taylor GI (1938). The spectrum of turbulence. Proc. R. Soc. A..

[54] Belen'kii, M., Karis, S., Osmon, C., Brown, J. & Fugate, R. *Experimental Evidence of the Effects of Non-Kolmogorov Turbulence and Anisotropy of Turbulence*. Vol. 3749 ICO (SPIE, 1999).

[55] Toselli I, Andrews LC, Phillips RL, Ferrero V (2008). Free-space optical system performance for laser beam propagation through non-Kolmogorov turbulence. Opt. Eng..

[56] Tsinober A (2013). The Essence of Turbulence as a Physical Phenomenon: With Emphasis on Issues of Paradigmatic Nature.

[57] Paul M (2010). Instabilities, Chaos And Turbulence.

[58] Shivamoggi BK (1998). Theoretical Fluid Dynamics.

[59] Biferale L, Musacchio S, Toschi F (2012). Inverse energy cascade in three-dimensional isotropic turbulence. Phys. Rev. Lett..

[60] Tsinober A (2018). The Essence of Turbulence as a Physical Phenomenon: With Emphasis on Issues of Paradigmatic Nature.

[61] Tsinober A (2009). An Informal Conceptual Introduction to Turbulence: Second Edition of An Informal Introduction to Turbulence.

[62] Dubrulle B (2019). Beyond kolmogorov cascades. J. Fluid Mech..

[63] She Z-S, Leveque E (1994). Universal scaling laws in fully developed turbulence. Phys. Rev. Lett..

[64] Perry A, Abell C (1975). Scaling laws for pipe-flow turbulence. J. Fluid Mech..

[65] Lancellotti RM, Vergara C, Valdettaro L, Bose S, Quarteroni A (2017). Large eddy simulations for blood dynamics in realistic stenotic carotids. Int. J. Numer. Method. Biomed. Eng..

[66] Mancini V, Bergersen AW, Vierendeels J, Segers P, Valen-Sendstad K (2019). High-frequency fluctuations in post-stenotic patient specific carotid stenosis fluid dynamics: A computational fluid dynamics strategy study. Cardiovasc. Eng. Technol..

[67] Yip KP, Holstein-Rathlou NH, Marsh DJ (1991). Chaos in blood flow control in genetic and renovascular hypertensive rats. Am. J. Physiol. Renal. Physiol..

[68] Yambe T (1994). Chaotic hemodynamics during oscillated blood flow. Artif. Organs.

[69] Bracic, M. & Stefanovska, A. in *Annual International Conference of the IEEE Engineering in Medicine and Biology—Proceedings.* 1740–1741.

[70] Bračič M, Stefanovska A (1996). Lyapunov exponents of quasi-periodic flows. Electrotech. Rev..

[71] Visee HF (1995). The physiological and clinical significance of nonlinear TCD waveform analysis in occlusive cerebrovascular disease. Neurol. Res..

[72] Ozturk, A. in *BIOSIGNALS 2016 - 9th International Conference on Bio-Inspired Systems and Signal Processing, Proceedings; Part of 9th International Joint Conference on Biomedical Engineering Systems and Technologies, BIOSTEC 2016.* 168–174.

[73] Ozturk A, Arslan A (2007). Classification of transcranial Doppler signals using their chaotic invariant measures. Comput. Methods Programs Biomed..

[74] Swinney, H. L. & Gollub, J. P. Hydrodynamic instabilities and the transition to turbulence. *Hydrodynamic instabilities and the transition to turbulence* (1981).

[75] Criminale WO, Jackson TL, Joslin RD (2018). Theory and Computation in Hydrodynamic Stability.

[76] Ginsberg JH (1973). The dynamic stability of a pipe conveying a pulsatile flow. Int. J. Eng. Sci..

[77] Straatman AG, Khayat RE, Haj-Qasem E, Steinman DA (2002). On the hydrodynamic stability of pulsatile flow in a plane channel. Phys. Fluids.

[78] Isler, J. A. & Carmo, B. S. in *Procedia IUTAM.* 580–589.

[79] Tsigklifis K, Lucey AD (2017). Asymptotic stability and transient growth in pulsatile Poiseuille flow through a compliant channel. J. Fluid Mech..

[80] Serrin J (1959). On the stability of viscous fluid motions. Arch. Ration. Mech. Anal..

[81] Freikamp A, Cost AL, Grashoff C (2016). The piconewton force awakens: Quantifying mechanics in cells. Trends Cell Biol..

[82] Epshtein M, Korin N (2017). Shear targeted drug delivery to stenotic blood vessels. J. Biomech..

[83] Sugiyama S (2016). Blood flow into basilar tip aneurysms: A predictor for recanalization after coil embolization. Stroke.

[84] Baratchi S (2017). Molecular sensors of blood flow in endothelial cells. Trends Mol. Med..

[85] Tovar-Lopez F (2019). A microfluidic system for studying the effects of disturbed flow on endothelial cells. Front. Bioeng. Biotechnol..

[86] Davies PF, Remuzzi A, Gordon EJ, Dewey CF, Gimbrone MA (1986). Turbulent fluid shear stress induces vascular endothelial cell turnover in vitro. Proc. Natl. Acad. Sci. USA.

[87] Davies PF, Remuzzi A, Gordon EJ, Dewey CF, Gimbrone MA (1986). Turbulent fluid shear stress induces vascular endothelial cell turnover in vitro. Proc. Natl. Acad. Sci..

[88] Ritman EL, Zamir M (2012). The Physics of Pulsatile Flow.

[89] A Matlab version of the Lyapunov exponent estimation algorithm of Wolf et al. Physica 16D, 1985. (Mathworks, https://www.mathworks.com/matlabcentral/fileexchange/48084-lyapunov-exponent-estimation-from-a-time-series-documentation-added, 2016).

[90] Schmid PJ, Henningson DS (2001). Stability and Transition in Shear Flows.

[91] Thomas TY (1941). Mathematical New Series.

[92] Thomas T (1943). On the uniform convergence of the solutions of the Navier-Stokes equations. Proc. Natl. Acad. Sci. USA.

[93] Hopf, E. in *Lecture Series of the Symposium on Partial Differential Equations, Berkeley.* 1–29.

[94] El-Gabry LA, Thurman DR, Poinsatte PE (2014). Procedure for determining turbulence length scales using hotwire anemometry.

