# Supplementary material for: Physiologic blood flow is turbulent
Source: Sci Rep. 2020 Sep 23;10:15492. doi: 10.1038/s41598-020-72309-8 (PMC7512016; doi:10.1038/s41598-020-72309-8)
Supplement: Supplementary file 1 — Supplementary information. [file 41598_2020_72309_MOESM1_ESM.docx]

**Physiologic Blood Flow is Turbulent**

**Supplementary Materials**

Khalid M. Saqr^1*^, Simon Tupin^1^, Sherif Rashad^2,3^, Toshiki Endo^3^, Kuniyasu Niizuma^2,3,4^, Teiji Tominaga^3^, Makoto Ohta^1^

^1^ Biomedical Flow Dynamics Laboratory, Institute of Fluid Science, Tohoku University, Sendai, 980-8577, Sendai 980-8577, Miyagi, JAPAN.

^2^ Department of Neurosurgical Engineering and Translational Neuroscience, Tohoku University Graduate School of Medicine, Sendai 980-8574, Miyagi, JAPAN.

^3^ Department of Neurosurgery, Tohoku University Graduate School of Medicine, Sendai 980-8574, Miyagi, JAPAN.

^4^ Department of Neurosurgical Engineering and Translational Neuroscience, Graduate School of Biomedical Engineering, Tohoku University, Sendai 980-8574, Miyagi, JAPAN.

**Corresponding author details:**

Khalid M. Saqr, Ph.D.

Biomedical Flow Dynamics Laboratory (Ohta-Lab)

Institute of Fluid Science, Tohoku University

Sendai, 980-8577, Sendai 980-8577, Miyagi, JAPAN

*Email: [k.saqr@tohoku.ac.jp](mailto:k.saqr@tohoku.ac.jp), [kh.saqr@gmail.com](mailto:kh.saqr@gmail.com)


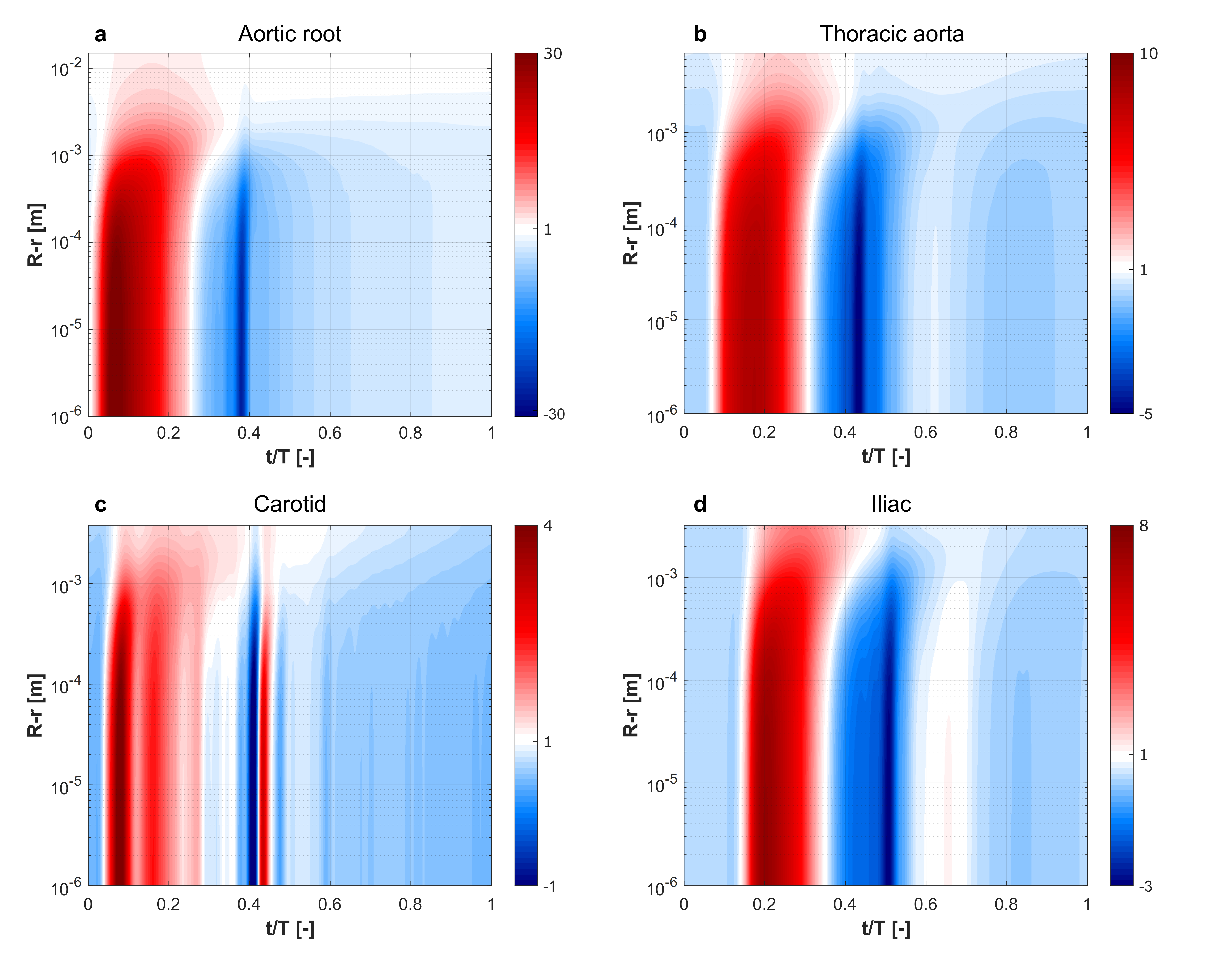


**Supplementary figure 1.** The ratio of fluctuation to mean velocity $\tilde{u}/U$ shown in colormaps for different arteries based on WFM with dimensionless time and radius in the X and Y axes, respectively.


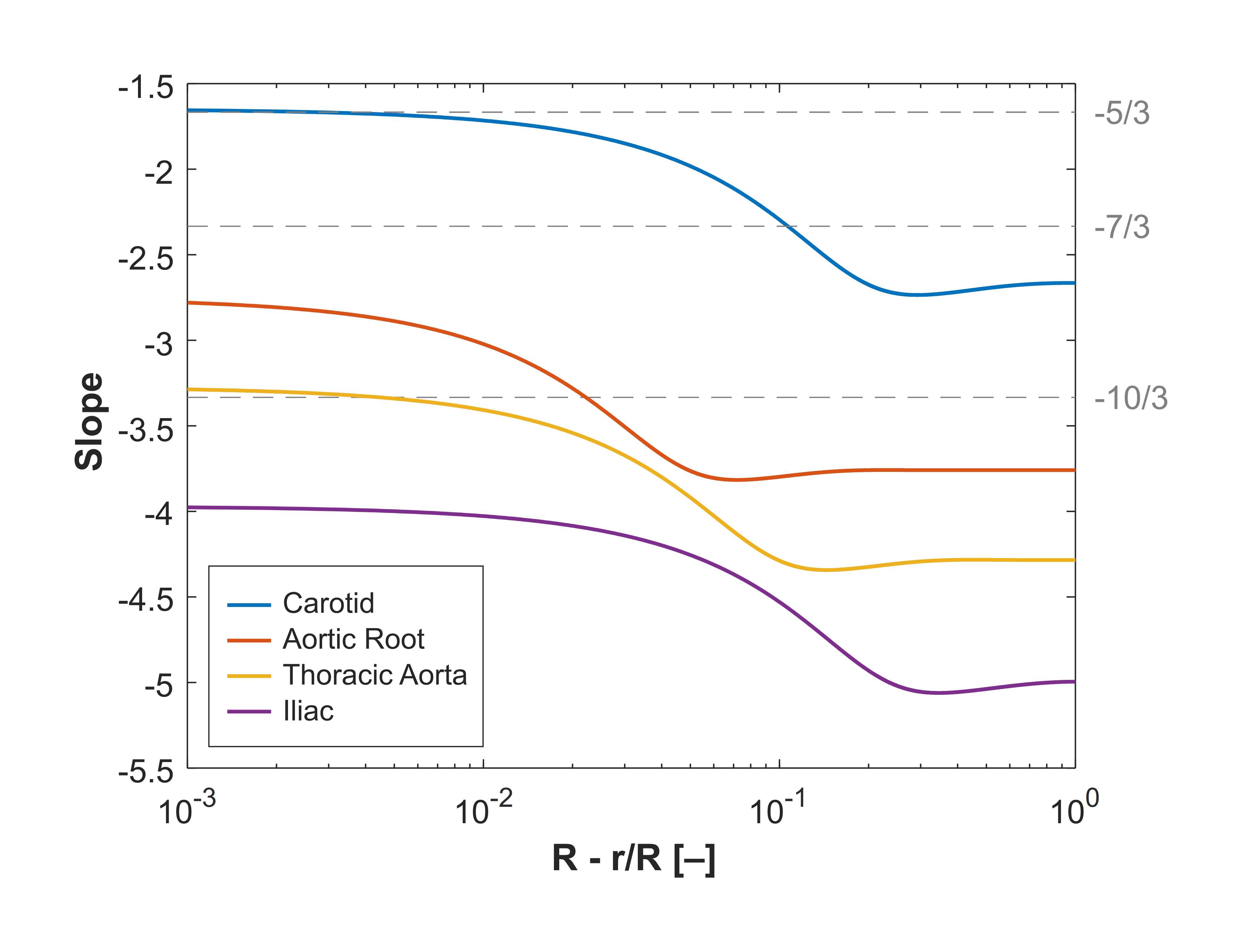


**Supplementary Figure 2.** Radial profiles of kinetic energy cascade slope of four arteries based on the exact solution of WFM.


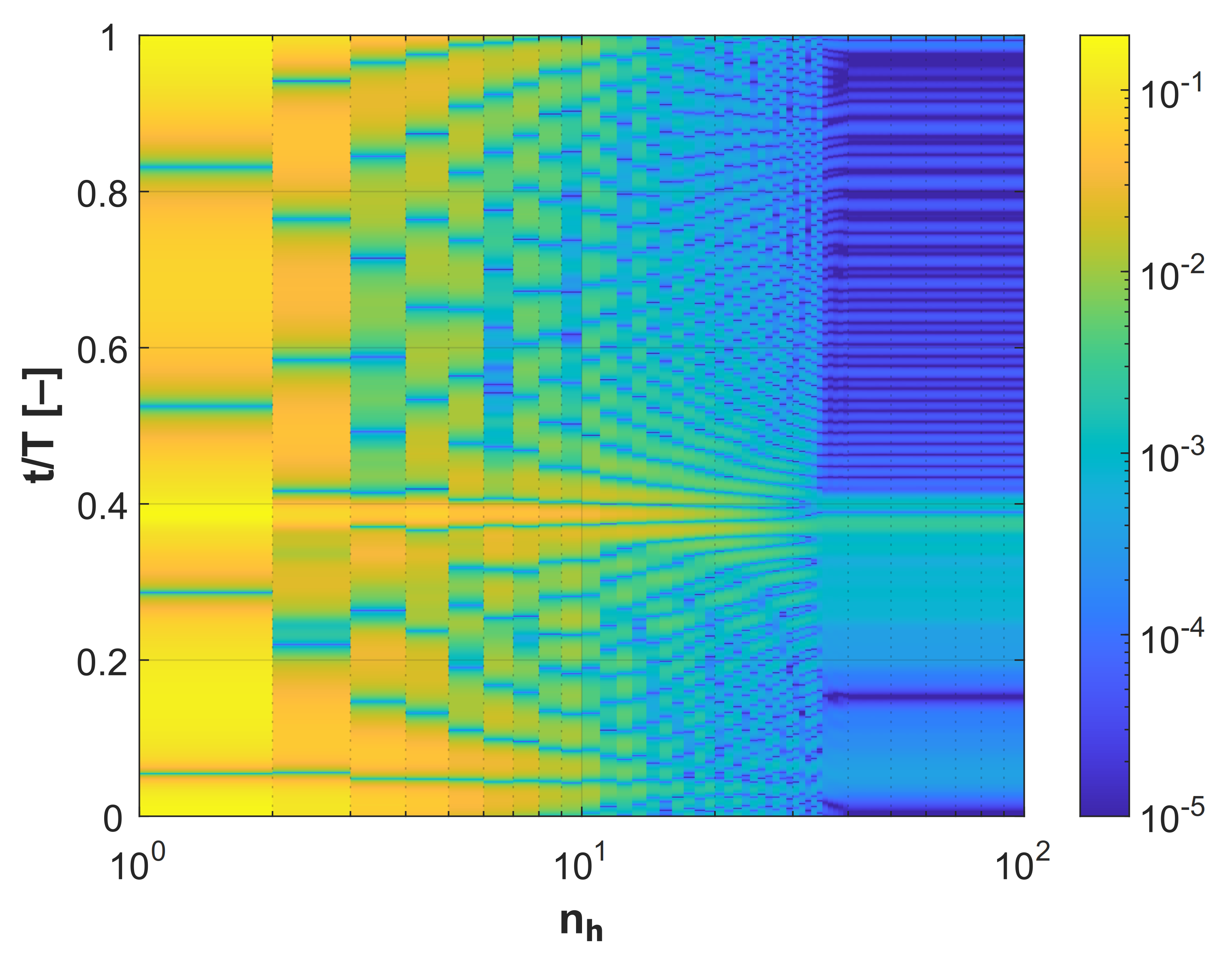


**Supplementary figure 3.** Color map of $\lim_{n\to100} \left| \frac{\dot{m}_{t}-\dot{m}_{[0:n]}}{\dot{m}_{t}} \right|$on x-axis and non-dimensional time on y-axis. (aortic root). Similar results were obtained from other arteries confirming that harmonics higher than $n=40$ could be neglected.
